# Multiphasic analysis of whole exome sequencing data identifies a novel mutation of ACTG1 in a nonsyndromic hearing loss family

**DOI:** 10.1186/1471-2164-14-191

**Published:** 2013-03-18

**Authors:** Gibeom Park, Jungsoo Gim, Arheum Kim, Kyu-Hee Han, Hyo-Sang Kim, Seung-Ha Oh, Taesung Park, Woong-Yang Park, ByungYoon Choi

**Affiliations:** 1 Departments of Biomedical Sciences, Seoul National University Graduate School, 110-799, Seoul, Korea; 2 Department of Otolaryngology, Seoul National University College of Medicine, 110-799, Seoul, Korea; 3 Interdiciplinary Program for Bioinformatics, College of Natural Science, Seoul National University, 151-742, Seoul, Korea; 4 Department of Statistics, College of Natural Science, Seoul National University, 151-742, Seoul, Korea; 5 Department of Otolaryngology, Seoul National University Bundang Hospital, 463-707, Seongnam, Korea; 6 Department of Molecular Cell Biology, Sungkyunkwan University School of Medicine, 135-710, Seoul, Korea; 7 Translational Genomics Laboratory, Samsung Genome Institute, Samsung Medical Center, 135-710, Seoul, Korea

**Keywords:** Hearing loss, Copy number variation, Linkage analysis, Single nucleotide variation, Mutation analysis

## Abstract

**Background:**

The genetic heterogeneity of sensorineural hearing loss is a major hurdle to the efficient discovery of disease-causing genes. We designed a multiphasic analysis of copy number variation (CNV), linkage, and single nucleotide variation (SNV) of whole exome sequencing (WES) data for the efficient discovery of mutations causing nonsyndromic hearing loss (NSHL).

**Results:**

From WES data, we identified five distinct CNV loci from a NSHL family, but they were not co-segregated among patients. Linkage analysis based on SNVs identified six candidate loci (logarithm of odds [LOD] >1.5). We selected 15 SNVs that co-segregated with NSHL in the family, which were located in six linkage candidate loci. Finally, the novel variant p.M305T in ACTG1 (DFNA20/26) was selected as a disease-causing variant.

**Conclusions:**

Here, we present a multiphasic CNV, linkage, and SNV analysis of WES data for the identification of a candidate mutation causing NSHL. Our stepwise, multiphasic approach enabled us to expedite the discovery of disease-causing variants from a large number of patient variants.

## Background

By virtue of the recent development of massively parallel DNA sequencing technologies, access to genomic composition has become easier than ever. With the advantage of exome sequencing, many studies have identified causal variants responsible for numerous disorders. Exome sequencing provides a particularly powerful method with which to identify disease-causing single nucleotide variations (SNVs) in Mendelian disorders [1-4]. Though whole exome sequencing (WES) has been used to successfully discover many genes that cause Mendelian disorders, analysis of WES data remains challenging [1]. An individual exome has more than 20,000 variants compared with the reference genome. Even in familial Mendelian disorders, the overall success rate for identifying disease-causing genes is around 50% [5]. Thus, many of the potential reasons for failure in the WES approach need to be solved fully in order to realize the promise of WES for routine diagnosis of Mendelian disorders.

Filtering patient data against normal populations and inferring identity-by-descent (IBD) in family studies can enrich candidate genes [4,6]. Genetic linkage analysis has also been a powerful tool for isolating potential causal candidate variants. A two-step approach of linkage analysis using single nucleotide polymorphism (SNP) microarrays to detect high logarithm of odds (LOD) score regions and subsequent targeted re-sequencing of regions of interest has been utilized in many genomic studies to intensify the power of detection [7]. Classically, microsatellite markers have been used for linkage analysis, and now millions of dimorphic SNP markers can be used to provide higher resolution in order to pinpoint candidate loci [8]. Currently, there are many efforts to use coding SNP information from WES data to facilitate genetic linkage mapping. Specifically, coding SNP data from WES can be used to establish multiphasic exome analyses based on linkages and SNVs [4,9].

Copy number variation (CNV) has been implicated in both Mendelian diseases [10] and common diseases such as obesity [11] and schizophrenia [12]. The presence of large insertions or deletions in patients is typically investigated prior to SNV analysis by karyotyping, fluorescence in situ hybridization (FISH), and/or array comparative genome hybridization (aCGH). Estimation of CNV is a challenging aspect of WES analysis, in which local depths of coverage must be mapped to copy numbers. Indeed, aCGH has limitations in detecting high CNV regions. Conversely, CNV data based on WES provides more accurate copy numbers because the depths of exon coverage from WES data vary linearly with real copy numbers [13]. Bioinformatics tools to analyze copy numbers from WES data are now publicly available [14].

Nonsyndromic hearing loss (NSHL) contributes to more than 70% of inherited cases of hearing loss. To date, approximately 50 genes have been shown to be causally related to NSHL. Many studies have identified more than 129 loci responsible for NSHL; however, 47 loci have not yet been mapped to proper genes [15,16]. The complexity of the auditory system may explain why so many genes and loci are linked to hearing loss. The genetic causes of hearing loss can be detected by sequence analysis, which helps clinicians and patients to delineate the basis of the disease. Given that hearing loss in early childhood can affect linguistic development [16], it is important to improve current techniques for identifying genetic alterations that cause NSHL. Earlier identification of such alterations in patients and families may allow for better clinical management of NSHL.

Analysis of WES data can be expanded to obtain more information useful for identifying causative mutations in Mendelian diseases. In this paper, in order to analyze WES data from an entire family, we applied three different methods, namely, CNV, linkage, and segregation analysis. By combining the results obtained from these methods, we efficiently identified a causative mutation from the family data. We applied this approach to WES data from a NSHL family to identify candidate disease-causing variants.

## Results

### Clinical features of a NSHL family

**Figure 1:**
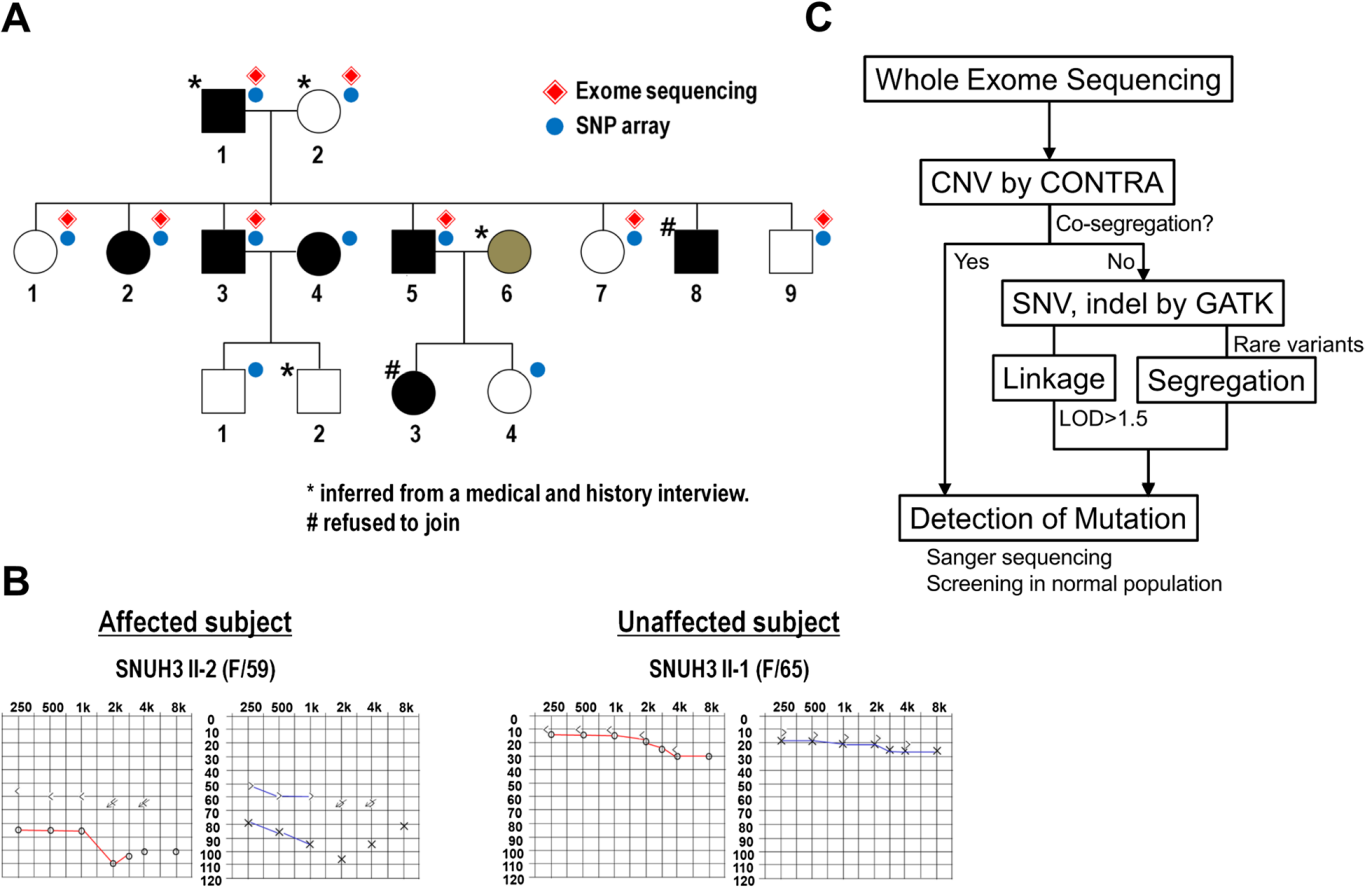
**NSHL family pedigree and clinical features.** Pedigree with phenotype and experimental information. Black and white denote affected and unaffected subjects, respectively, while shaded grey represents equivocal hearing status (**A**). Typical audiograms of affected and unaffected subjects (**B**). Scheme of the multiphasic analysis of WES data (**C**).

We identified a Korean family with six members affected by NSHL and seven unaffected members (Figure 1A). Pure tone audiometry (PTA) was performed on nine family members, three of whom (II-2, II-3, and II-5) exhibited profound post-lingual hearing loss. The three members had normal cognitive function and no anomalous-looking features. They went through a battery of clinical tests ranging from general physical examinations, chest X-rays, and simple blood tests to detailed imaging studies including brain MRI and temporal bone CT scans. No abnormalities were detected in the tests, excluding the possibility that hearing loss in these patients was syndromic. The other siblings (II-1, II-7, and II-9) exhibited normal hearing (Figure 1B). Patients II-2, II-3, and II-5 estimated that their hearing loss became severe in their 30’s, during which time they started to wear hearing aids. Their hearing loss was further aggravated and became profound in their late forties. Ultimately, patients II-3 and II-5 no longer benefited from hearing aids and underwent cochlear implantation. They achieved recognition of common sentences without lip reading one year after implantation. *GJB2* is one of the most frequently detected genes in individuals with NSHL, and thus we first investigated the sequence of *GJB2* in the NSHL patients. After failing to identify any mutations in *GJB2*, we performed WES on several members of the Korean family in order to identify a disease-causing mutation.

### Copy number analysis using WES data

**Figure 2:**
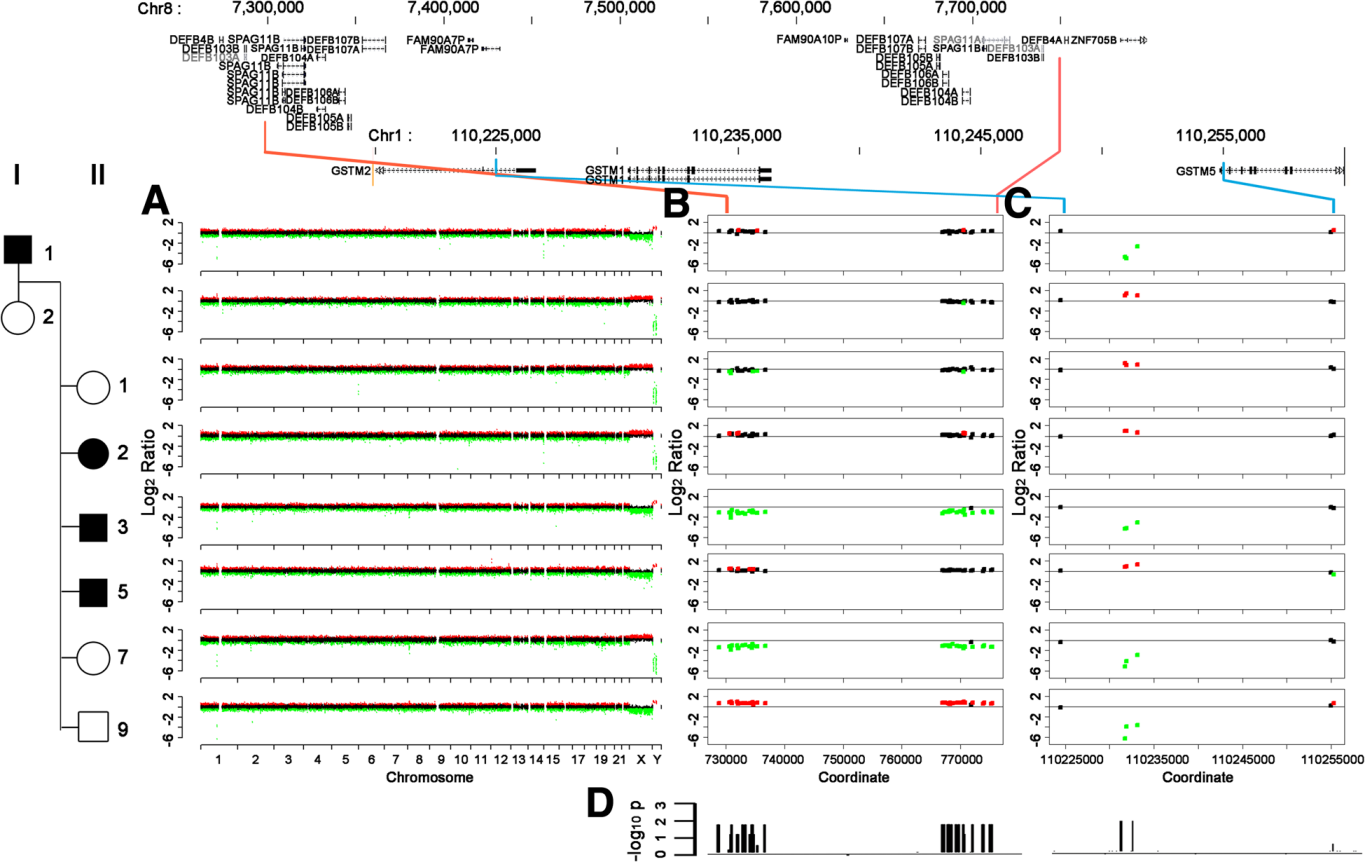
**CNV detected by WES.** CNV throughout the chromosomes – 1p13.3, 4q13.2, 5q35.3, 8p23.1, and 19q13.4 have distinct CNVs (14q32.3 is distinct, but contains variable regions associated with antibody production) (**A**), 8p23.1 containing beta-defensin clusters (**B**), and 1p13.1 containing *GSTM1* (**C**) of eight subjects. Red and green dots are exons with p<0.05. Co-segregated regions of CNVs were also analyzed by Fisher’s exact test (**D**).

WES data was obtained from the parents and six siblings (four affected and four unaffected members, Figure 1A). The mean coverage of each sample ranged from 40.3X to 51.3X, and 87.0% to 90.5% of the targeted exome had at least 10 reads. A multiphasic WES analysis was designed to find a causative NSHL mutation (Figure 1C). First, we investigated co-segregation of copy number duplication or deletion in exomes of patients using CONTRA software. We detected five CNV loci with distinct features in the plots (Figure 2A). None of the CNVs co-segregated with affected or unaffected family members. One CNV locus of the CNVs from three members (high copy number exons in II-9, and low copy number exons in II-3 and II-7) was located in 8p23.1, a region that contains beta-defensin genes and *SPAG11* (Figure 2B). The following genes were identified as being located at regions of distinct CNVs in the indicated family members: *GSTM1* in 1p13.3 (I-1, II-3, II-7, and II-9) (Figure 2C), *UGT2B17* in 4q13.2 (I-2 and II-7), *BNTL3* in 5q35.3 (II-1), and *LILRB2* in 19q13.4 (I-2) (data not shown). We also applied Fisher’s exact test for the LOD score per exon to detect co-segregated regions of CNVs, but there were no peaks with values reaching significance. We identified two groups based on the pattern of segregation of *SPAG11, GSTM1,* and beta-defensin genes to validate the relevance of this method (Figure 2D).

### Exome linkage analysis

**Figure 3:**
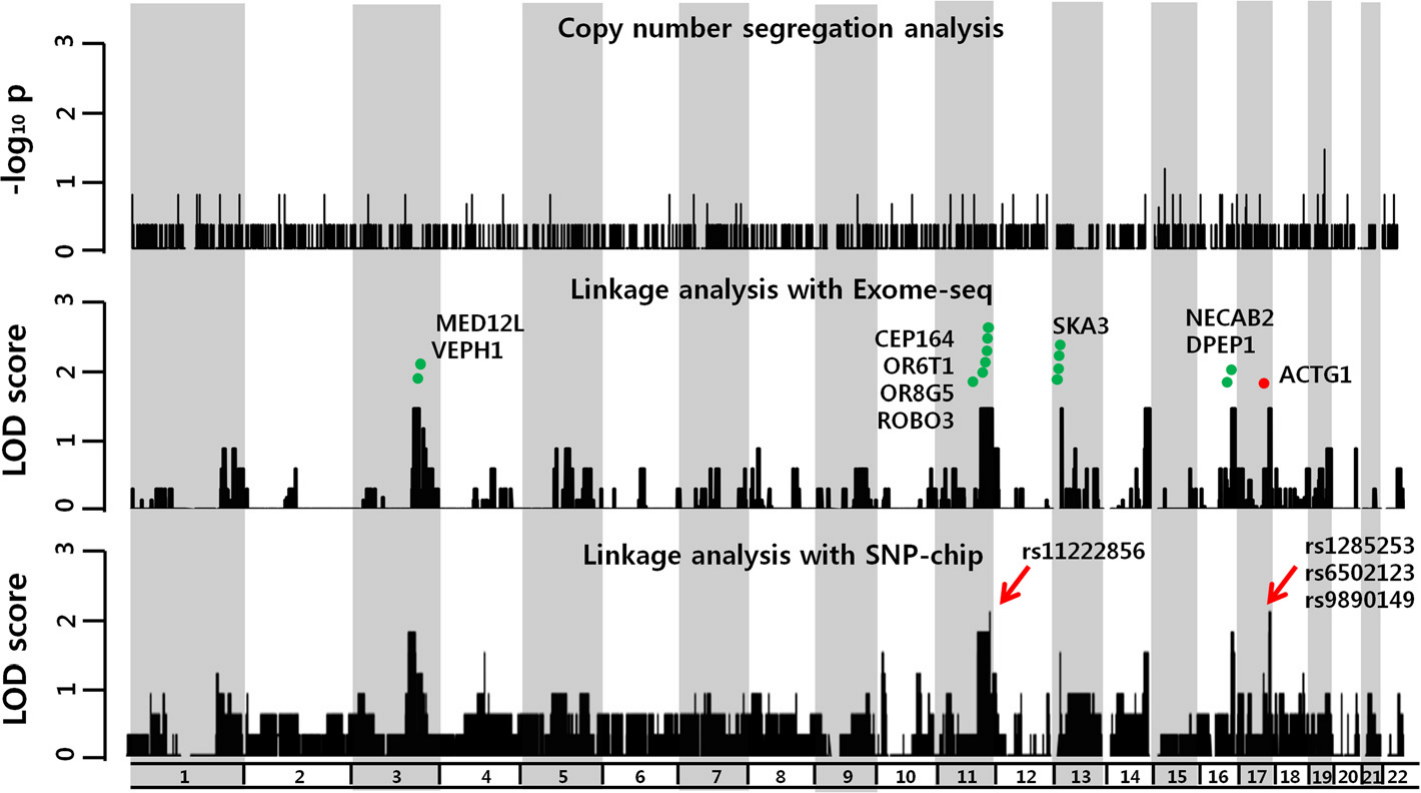
**A multiphasic analysis of WES data.** WES data were analyzed for exon CNVs and SNVs. Fisher exact test on CNVs detected one exon segregating with NSHL on chr19 (top). Linkage analysis with SNVs called by Exome-seq identified six disease-linked “hot spots” on chr3, chr11, chr13, chr14, chr16, and chr17 (middle). Segregation analysis independently identified 15 SNVs co-segregating with NSHL (green dots). Among them, a novel variant resulting in p.M305T, in ACTG1 on chr17 was validated with Sanger sequencing (red dot). Linkage analysis was also performed with SNP microarray by adding three more subjects in the family. Not only were similar “hot spots” detected, adding more subjects in the analysis enhanced the true peak (red arrow) (bottom).

Because the pedigree strongly suggested an autosomal dominant mode of inheritance, we identified 17,498 coding autosomal SNVs from WES data and performed single-point linkage analysis. We identified six hot spots where a number of peaks were closely clustered (Figure 3). Specifically, we identified peaks on chromosomes 3, 11, 13, 14, 16, and 17 consisting of 11, 67, 2, 13, 17, and 13 exons, respectively.

We validated single-point linkages using a SNP microarray containing 328,125 SNPs. Along with the eight initial family members recruited for WES analysis, we included three additional subjects (II-4, III-1, and III-4) to validate the significance of peaks obtained from exome linkage analysis. The six hot spots detected from sequencing data were also detected in microarray analysis with a relatively high LOD score (Figure 3). Adding three more subjects to the linkage analysis enhanced the peaks at chromosomes 11 and 17, which consisted of one and three SNPs (LOD score >2), respectively. The genotype patterns of these four peaks were perfectly matched with an autosomal dominant mode of inheritance.

### SNV analysis

**Figure 4:**
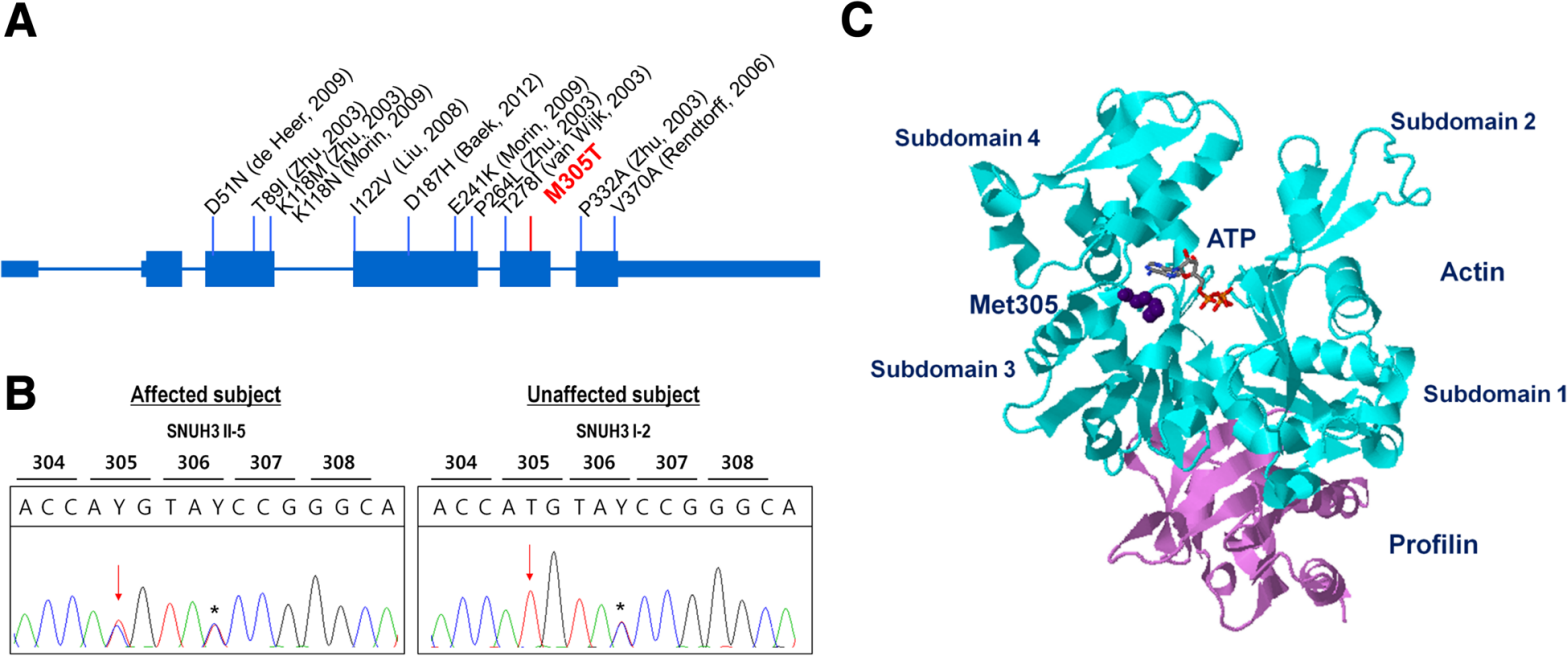
**p.M305T mutation in ACTG1.** The p.M305T mutation reported in this study as well as several other previously reported mutations in ACTG1 cause hearing loss (**A**). p.M305T (arrow), confirmed by Sanger sequencing, co-segregated perfectly with hearing loss (asterisk: synonymous SNV) (**B**). Met305 is located close to the ATP binding site of ACTG1 (**C**).

Based on the WES analysis of four affected and four unaffected family members, we identified 18,748~20,025 SNVs and 413~457 indels. These were reduced to 962~1,123 SNVs and 140~153 indels after filtering through the dbSNP135 and 1000 Genome databases. Fifteen variants causing amino acid changes were selected based on their co-segregation pattern within the family (Table 1). All of the 15 variants on chromosomes 3, 11, 13, 16, and 17 corresponded to regions with high LOD scores (Figure 3). One novel mutation in actin gamma 1 (ACTG1) was identified, consisting of a methionine to threonine substitution at amino acid 305 (p.M305T), This candidate variant was validated by Sanger sequencing and co-segregated with hearing loss in all family members (Figure 4A and B).

**Table 1:** Nonsynonymous SNVs and indels identified in patients but not in non-symptomatic family members

**Gene**	**Chr**	**Nucleotide variation**	**Amino acid variation**	**Frequency in 1,000 genome**	**dbSNP135**
MED12L	chr3	c.G3629A	p.R1210Q	0.23	rs3732765
VEPH1	chr3	c.T1564C	p.S522P	0.28	rs11918974
CWF19L2	chr11	c.A2681G	p.Y894C	0.27	rs3758911
CEP164	chr11	c.G281A	p.S94N	0.19	rs490262
OR6T1	chr11	c.G465C	p.W155C	0.0046	rs150534954
OR8G5	chr11	c.G287A	p.C96Y	0.45	rs2512168
OR8G5	chr11	c.G716A	p.G239E	0.50	rs2512167
ROBO3	chr11	c.G1247A	p.R416H	0.14	rs3862618
SKA3	chr13	c.A1157G	p.K386R	0.13	rs11147976
SKA3	chr13	c.C1142T	p.T381I	0.11	rs11147977
SKA3	chr13	c.G559A	p.V187I	0.14	rs61950353
SKA3	chr13	c.208delC	p.Q70fs	-	rs151272242
NECAB2	chr16	c.C704G	p.T235S	0.20	rs2292324
DPEP1	chr16	c.G1051C	p.E351Q	0.24	rs1126464
ACTG1	chr17	c.T914C	p.M305T	-	-

ACTG1 (DFNA20/26; MIM: 604717) was strictly conserved in 19 of 20 eukaryotes analyzed (HomoloGene:74402), with the M305 codon being conserved in 19 species. Protein damage prediction analysis identified p.M305T as “possibly damaging” by HumDiv, “probably damaging” by HumVar in Polyphen2 [17], and “disease causing” by MutationTaster [18]. The mutation site, Met305, was visualized using the 3D structure of bovine beta-actin bound by adenosine triphosphate (ATP) with profilin (Figure 4C). The methionine was closely located to the ATP molecule. Additionally, Met305 is listed as a predicted residue for the ATP binding site by the Protein Data Bank (PDB).

## Discussion

WES is a powerful technique that can be used to discover causative genes in human diseases. Although WES has been integral in identifying more than 1,000 novel genes in Mendelian disorders [1], there is still a need for increased efficiency of gene discovery using WES data. In this regard, we analyzed WES data from a family with a history of NSHL by focusing on three categories of genetic information: CNVs, linkage analysis, and SNVs. Utilizing these data, we undertook a stepwise multiphasic approach to identify disease-causing variations in the family.

8p23.1, which contains a beta-defensin cluster, was detected as a region with high copy number (II-9) and low copy number (II-3 and II-7) (Figure 2). The defensin cluster, containing both alpha- and beta-defensins, was previously studied as a dynamic genomic region with varying copy numbers ranging from one to twelve [19]. The parents had normal copy numbers, which was in contrast to the low copy numbers seen in two children and high copy number observed in one child. A total of four haplotypes of 8p23.1 may have been inherited in this family, and each parent may have had both under- and over-amplified alleles of 8p23.1. The overall copy number of a parent can appear to be normal due to compensation of copy number from over- to under-amplified alleles [20]. In the family in this study, *GSTM1* and *UGT2B17,* genes with frequently reported deletions [21], as well as *BNTL3* and *LILRB2,* exhibited CNVs. We used Fisher’s exact test on the affected and unaffected family members after validating this method for 8p23.1 and *GSTM1* groups to determine the amplification or deletion of multiple exons that matched the co-segregation pattern of the disease. Multiple statistically significant peaks at 8p23.1 and *GSTM1* were identified, and were identical to plots from the first approach. However, there was only one statistically significant peak identified by testing the two groups that segregated with the disease, and this peak did not correlate with disease status. Thus, while WES may provide a method to identify CNV regions with highly similar sequences, determining accurate copy numbers can prove difficult.

Linkage analysis was performed to narrow down the number of candidates based on WES data. Importantly, linkage analysis with a relatively small number of markers still provides useful information. Fewer markers from WES data are available and can be obtained from a SNP microarray, and the markers that are identified may not be evenly distributed. Given these limitations, it is necessary to consider the potential disadvantages of this approach. Because we analyzed only exonic SNPs (~1% of genome-wide SNPs), we may have lost critical information located outside of exons. In addition, potential genotyping errors in linkage analyses can reduce statistical power for detecting linkage peaks or result in false positive linkage peaks [22]. Even so, the results obtained from the different data sets in this study confirmed the validity of our approach. Linkage analysis requires a large number of subjects to help identify putative loci. Unless a proper number of subjects are available, an informative result is difficult to obtain.

After applying linkage analysis results, the co-segregated variants were all found to be located in the loci of high LOD scores. However, linkage analysis can decrease the number of candidate variants, particularly in instances where candidate variants are widely distributed. Additional linkage analysis of WES data demonstrated a similar performance to that of SNP microarray data and simultaneously generated results during variant calling. Considering that CNVs could be also detected using this approach, the multiphasic analysis of WES data efficiently narrowed and identified candidate variants and was advantageous compared with established methods such as initial aCGH, variant calling according to WES data alone, or linkage analysis based on SNP microarray data.

Actin is a highly conserved cytoskeletal protein that plays important roles in eukaryotic cell processes such as cell division, migration, endocytosis, and contractility. Actin isoforms are classified into two groups based on expression patterns. ACTA1, ACTA2, ACTC, and ACTG2 are “muscle” actins, predominantly expressed in striated or smooth muscle, whereas ACTB and ACTG1 are cytoplasmic “non-muscle” actins [23]. Autosomal dominant progressive sensorineural hearing loss, DFNA20/26 (MIM: 604717), is caused by a mutation in the gamma-actin gene on chromosome 17 at q25.3. Some ACTG1 mutations are associated with Baraitser-Winter syndrome, which is characterized by developmental delay, facial dysmorphologies, brain malformations, colobomas, and variable hearing loss. The constellation of these abnormalities is suggested as the most severe phenotype of ACTG1 mutations [24,25]. A genome-wide screen of DFNA20 localized candidates to 17q25.3 [26] and mapped the causative missense mutations to highly conserved actin domains of the gamma-actin gene (*ACTG1*) [27,28]. *In vivo* and *in vitro* studies of ACTG1 indicate that it is required for reinforcement and long-term stability of actin filamentous structures of stereocilia, but not for auditory hair cell development, which is in line with the progressive nature of hearing loss related to *ACTG1* mutations in humans [29,30]. Further, missense mutations in either *ACTB* or *ACTG1* have recently been reported to cause Baraitser-Winter syndrome. Interestingly, of the 11 mutations that cause DFNA20 [27,28,31-34] and 6 mutations that cause Baraitser-Winter syndrome (see OMIM entry - *102560) that have been reported, are all missense mutations. The predicted interaction between Met305 and ATP in bovine beta-actin, a protein with a 99% identity to ACTG1, implies that the mutation of Met305 may influence ATP binding of ACTG1, which is essential for polymerization of G-actin to F-actin.

ACTG1 is predominantly expressed in intestinal epithelial and auditory hair cells [35]. Detection of exclusively missense mutations in this gene may imply that truncating mutations have more severe effects and might cause embryonic lethality. The hearing impaired subjects in this study (II-2, II-3, and II-5) did not report any gastrointestinal complaints. The subjects in this study required cochlear implants, recapitulating what has previously been reported regarding the management of patients with mutations in *ACTG1* and resultant NSHL [31]. The severe phenotype and rapid progression of hearing loss to a profound level within one or two decades associated with mutations in *ACTG1* necessitates an early molecular genetic diagnosis and timely auditory rehabilitation.

## Conclusions

Two or more platforms (aCGH, SNP array, and WES) have previously been required to generate complex genetic information such as CNVs, linkages, SNVs, and indels. In general studies of Mendelian disorders, WES has primarily been utilized to obtain only SNVs and indels. Our study agrees well with other work demonstrating that analysis of WES data also allows for CNV and linkage determination due to its quantitative traits. Given the robust nature of WES data, it is clear that the full capabilities of this relatively new technology have not yet been fully realized. Our multiphasic WES analysis proved very powerful for the interpretation and narrowing of WES results, in particular when a large amount of family data is available.

## Methods

### Subjects

This study was approved by the Institutional Review Boards (IRBs) of Seoul National University Hospital (SNUH) and Seoul National University Bundang Hospital (SNUBH). Written informed consent for participation in the study was obtained from participants or from a parent or guardian in the case of child participants. A three generation pedigree was established for the family (SNUH3) (Figure 1A). Among the 15 subjects in the SNUH3 family, 13 were willing to participate in this genetic study, while two reportedly deaf subjects (II-8 and III-3) refused participation. DNA from blood lymphocytes was isolated from the 12 subjects, while DNA from III-2 was obtained with a buccal swab.

### Audiometric analysis

Pure tone and speech audiometry and physical examinations were performed for nine members of the cohort (Figure 1B). Pure tone audiometry (PTA) with air and bone conduction at frequencies ranging from 250 to 8,000 Hz was obtained from the recruited subjects according to standard protocols. The auditory phenotype was inferred from thorough medical and developmental history interviews from one deaf subject (I-1), two likely unaffected subjects (I-2 and III-2), and one subject (II-6) with an equivocal hearing status.

### WES

Eight of the 13 recruited subjects (four affected and four unaffected) were chosen for commercial WES (Otogenetics, Norcross, GA) and analyzed as previously reported [3]. Briefly, paired-end reads of 100 bp from the eight subjects were aligned by bwa-0.6.1 to the UCSC hg19 reference genome using default settings. SAMtools and Picards were used to process SAM/BAM files and mark duplicates. Local realignment around indels and base quality score recalibration was done for each sample, and variants were called by a unified genotyper in GATK-1.3. Perl scripts and ANNOVAR were used to annotate variants and search the relevant known SNPs and indels from dbSNP135 and the 1000 Genome database. Variants with a read depth greater than 10 and genotype quality score greater than 30 were filtered for further analysis.

### CNV analysis using WES data

CNVs were detected by CONTRA software [36] using BEDTools to calculate coverage per exon and apply statistics to normalize coverage data and test fold changes. A new baseline file was produced using our data, but we expected to detect distinct deletions or amplifications. Polymerase chain reaction (PCR) duplicates were removed by Picards before using CONTRA.

We tabulated a 3 × 2 exon copy variation contingency table based on the whole per-exon CNV status of the eight subjects (Table 2). Fisher’s exact test was used to assess the significance of differences between proportions of abnormal copy number events present in affected and unaffected family members. We assumed that all of the subjects were independent in order to conduct an alternative practical method to find loci that segregated with the disease.

**Table 2:** Exon copy number variation contingency based on the whole per-exon CNV status

**Copy number**	**Patient**	**Normal**	**Total**
gain	n_21_	n_20_	n_2+_
normal	n_11_	n_10_	n_1+_
loss	n_01_	n_00_	n_0+_
	n_+1_	n_+0_	8

### Linkage analysis using WES and SNP microarray data

Using WES data, we filtered out the following variants: those located on sex chromosomes, those with low coverage (<10X), and those with a low genotype quality score (<30) in any of the eight subjects with 17,498 SNVs. We used a Genome-Wide Human SNP Array 6.0 (Affymetrix, Santa Clara, CA), which contains 328,125 SNP markers located on autosomal chromosomes. We performed parametric linkage analysis with the R package paramlink [37]. The pedigree suggested an autosomal dominant mode of inheritance, and thus we assumed an autosomal dominant model with default values of full penetrance (f_0_, f_1_, f_2_) = (0, 1, 1) and disease allele frequency = 1e-05. The penetrance parameters f_0_, f_1_, and f_2_ were also defined using conventional notation as below.

$$f_{i}=P\left(\mathrm{affected}\;|i\;\mathrm{copies}\;\mathrm{of}\;\mathrm{the}\;\mathrm{disease}\;\mathrm{allele}\right)$$

The recombination fraction between the disease locus and markers was set to θ=0 by default. We computed single-point LOD scores for all markers. We compared LOD scores from SNP microarray and WES. We matched the subjects and the markers that were common between both platforms using manual python and R scripts. Finally, single-point analyses were performed with all of the data.

### 3D structure of actin gamma-1 (ACTG1)

Protein damage prediction analysis was performed using HumDiv and HumVar in Polyphen2 [17], and also by MutationTaster [18]. The mutation site was visualized using the 3D structure of bovine beta-actin bound by adenosine triphosphate (ATP) with profilin. Bovine beta-actin actin has a 99% identity with human gamma-actin. The ATP binding site was analyzed using the Research Collaboratory for Structural Bioinformatics (RCSB) Protein Data Bank (PDB) ( http://www.pdb.org) [38]. PDB entry 2BTF [39] on P60712 (ACTB_BOVIN) with P02584 (PROF1_BOVIN) was downloaded, visualized, and modified by Bioclipse [40] to observe the 305Met residue in 3D.

## Competing interests

The authors declare that they have no competing interests.

## Authors’ contributions

GP participated in the design of the study, analysis and interpretation of data; JG analyzed copy number variation from the exome data; AK validated variants by Sanger sequencing and checked segregation of the variant; KH collected samples from the family; HK analyzed clinical features; SO collected patients and initiated this study; TP have been involved in drafting the manuscript; WP designed the study, wrote the manuscript, revised it; BC participated in the design of the study, interpretation of data, and drafting the manuscript, revising it critically and have given final approval of the version to be published. All authors read and approved the final manuscript.
